# Gait Assessment Using Smartphone Applications in Older Adults: A Scoping Review

**DOI:** 10.3390/geriatrics9040095

**Published:** 2024-07-18

**Authors:** Lorenzo Brognara

**Affiliations:** Department of Biomedical and Neuromotor Sciences (DIBINEM), University of Bologna, 40123 Bologna, Italy; lorenzo.brognara2@unibo.it

**Keywords:** aging, frailty, gait, smartphone, mobile app

## Abstract

Spatiotemporal parameters such as gait velocity and stride length are simple indicators of functional status and can be used to predict major adverse outcomes in older adults. A smartphone can be used for gait analysis by providing spatiotemporal parameters useful for improving the diagnosis and rehabilitation processes in frail people. The aim of this study was to review articles published in the last 20 years (from 2004 to 2024) concerning the application of smartphones to assess the spatiotemporal parameters of gait in older adults. This systematic review was performed in line with Preferred Reporting Items for Systematic Review and Meta-Analyses (PRISMA), and original articles were identified by searching seven electronic databases: SciVerse (ScienceDirect), Excerpta Medica Database (EMBASE), Medline, Scopus, PubMed, Web of Science and the Cochrane Library. Studies were rigorously screened using the inclusion criteria of smartphones and mobile apps, older adults and spatiotemporal gait parameters, and results were narratively synthesized. Seventy-three articles were initially identified while searching the scientific literature regarding this topic. Eleven articles were selected and included in this review. Analysis of these studies covered information about gait assessment using mobile apps recorded in 723 older adults and 164 control cases. Analysis of data related to the application of smartphones to assess spatiotemporal parameters of gait in older adults showed moderate-to-excellent test–retest reliability and validity (ICCs around 0.9) of gait speed, the most common parameter reported. Additionally, gait speeds recorded with mobile apps showed excellent agreement when compared to gold standard systems. Smartphones and mobile apps are useful, non-invasive, low-cost and objective tools that are being extensively used to perform gait analysis in older adults. Smartphones and mobile apps can reliably identify spatiotemporal parameters related to adverse outcomes, such as a slow gait speed, as predictors and outcomes in clinical practice and research involving older adults.

## 1. Introduction

Numerous studies have analyzed gait in older adults and its relationship with adverse outcomes, including disability, dementia, hospitalization and mortality [1,2,3,4]. Different spatiotemporal variables related to adverse outcomes have been identified in the scientific literature, such as gait speed, stride length and cadence [5]. Older adults who present a slower gait and a decrease in stride length are considered patients at risk. In particular, a gait speed lower than 0.8 m/s is a reliable cut-off for identifying subjects at increased risk of disability, and a stride length of 0.64 m accurately predicts major adverse events such as physical disability, falls, institutionalization and mortality [6,7]. With the advancement of medical and health standards, the proportion of people over 60 accounts for 12.3% of the global population, and World Health Organization studies show that by 2050 this figure will approximately double from 12% to 22% [8,9,10]. Aging is associated with physiological changes that can reduce mobility and quality of life in people aged 65 years and older, but age-related gait decline is still underdiagnosed or diagnosed at a relatively late stage, often following an injury, despite increasing evidence suggesting that a decrease in walking speed represents a key element of frailty [11]. Over the last decade, several reports have pinpointed the importance of early detection in patients with gait impairments and those at risk of falling [12,13]. The use of smartphone apps in daily life may be adapted to the early detection of older individuals with a reduced walking speed and can help identify individuals who are at higher risk of falling before an episode of fall or an advanced stage of dementia [14,15]. Smartphone apps are widely used to collect data on gait parameters such as cadence, gait speed and step length, enabling monitoring of the patient’s status in a free-living environment [16]. Older adults are increasingly using smartphones and mobile apps, which may serve as useful tools to support gait assessment [17,18]. Early detection of a decline in gait parameters may help older adults adopt timely interventions in order to improve their quality of life and predict adverse outcomes [19]. The aim of this scoping review was to provide current evidence regarding the effectiveness of gait assessment using a mobile app on a smartphone in the aged population, and results of this study highlight the importance of gait impairments and enhance our knowledge of gait assessment protocols as a simple indicator to predict major adverse outcomes in older adults.

## 2. Materials and Methods

### 2.1. Identifying the Research Question

The primary aim of this review was to synthesize all the published evidence on gait impairment evaluated with smartphones in older adults. The review question was formulated using the PCC strategy (population, concept and context), and eligibility criteria were developed based on populations, interventions or exposures, comparators, outcomes and study designs (PICOS): participants: older adults (over the age of 65 years); interventions or exposures: not applicable; comparators: data collected with a smartphone and/or mobile app; outcomes: gait assessment; study designs: cross-sectional and prospective cohort observational studies and intervention studies. The review question was as follows: are smartphone-based gait assessments in older adults a reliable method of quantifying the spatiotemporal parameters of gait and predicting adverse outcomes? The goal of this work was to provide an overview of the recent state of the art in this field, characterizing current usage and limitations.

### 2.2. Literature Search Methodology

The following systematic review was performed in line with Preferred Reporting Items for Systematic Review and Meta-Analyses (PRISMA). The research question was developed using the PICO framework. This study analyzed every original article published up to April 2024 that met the following inclusion criteria: (1) full text in English; (2) primary articles only; and (3) presentation of identifiable data measuring gait with a smartphone in older adults. Studies were searched in the following databases: Scopus, Excerpta Medica Database (EMBASE), Medline, SciVerse (ScienceDirect), PubMed, Web of Science and the Cochrane Library. The search strategy was formulated based on a combination of controlled descriptors and keywords related to the topic. Moreover, a manual search was conducted of reference lists from initially selected studies to identify other eligible studies.

Conference proceedings and articles reporting results from fewer than twenty older patients that did not assess gait or that assessed only balance were excluded. Articles were excluded if they focused on mobility assessment for older people with neurological disorders such as Alzheimer’s disease or Parkinson’s disease.

An electronic search was performed in PubMed on 11 April 2024 using the following search string:

(“smartphone”[MeSH Terms] OR “smartphone”[All Fields] OR “smartphones”[All Fields] OR “smartphone s”[All Fields]) AND (“gait”[MeSH Terms] OR “gait”[All Fields]) AND (“aged”[MeSH Terms] OR “aged”[All Fields] OR (“older”[All Fields] AND “adults”[All Fields]) OR “older adults”[All Fields]).

(“mobile applications”[MeSH Terms] OR (“mobile”[All Fields] AND “applications”[All Fields]) OR “mobile applications”[All Fields] OR (“mobile”[All Fields] AND “app”[All Fields]) OR “mobile app”[All Fields]) AND (“gait”[MeSH Terms] OR “gait”[All Fields]) AND (“aged”[MeSH Terms] OR “aged”[All Fields] OR (“older”[All Fields] AND “adults”[All Fields]) OR “older adults”[All Fields]).

**Figure 1:**
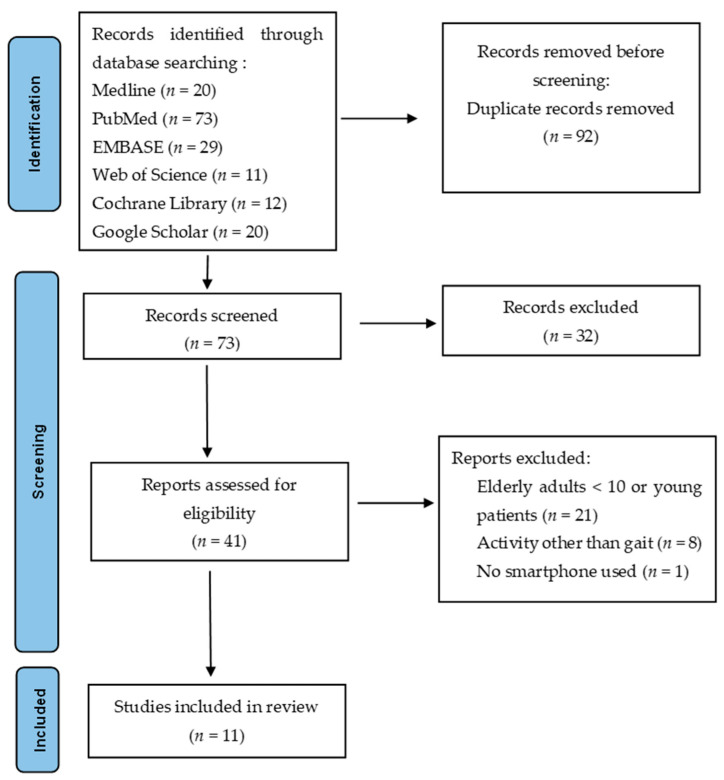
PRISMA diagram: this figure represents the flow of study selection through identification, screening, eligibility and inclusion.

A number of articles (165) were initially identified while searching the scientific literature regarding this topic, and 11 articles were selected and included in this review. Figure 1 presents a flowchart of the review process (PRISMA diagram).

Finally, reference lists of all relevant articles were manually cross-referenced in order to identify any additional articles. A qualitative synthesis of data from selected studies was conducted, describing the following data: (1) characteristics of the studies (name of the study, authors, year of publication); (2) demographic information for samples (sample size, participant characteristics of mean age, gender distribution); (3) spatiotemporal parameters investigated; (4) mobile app used; (5) smartphone used and (6) main findings. These data are summarized in Table 1, which lists characteristics of mobile apps, smartphones used and spatiotemporal parameters.

## 3. Results

**Figure 2:**
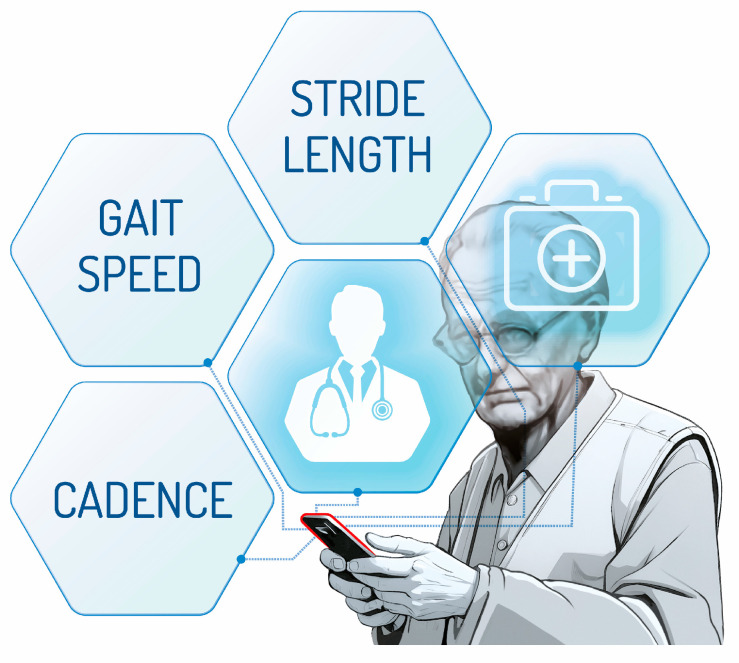
Spatiotemporal parameters most frequently evaluated in selected articles.

Studies included an average of 74 older patients with a sample of at least 28 participants [14] and a maximum of 163 participants [23]. The minimum average age observed in these studies was 56.12 ± 6.06 years [21], with a maximum of 75.6 years [22]. These studies had a specific sex target; in most studies, women were the most prevalent. Concerning gait spatiotemporal parameters, substantial variety was found in selected articles. Gait speed and step length are the most common parameters reported in these studies (Figure 2). Cadence, which can be particularly useful when evaluating older patients (who often may exhibit short steps), is reported in only two studies [23,27].

**Figure 3:**
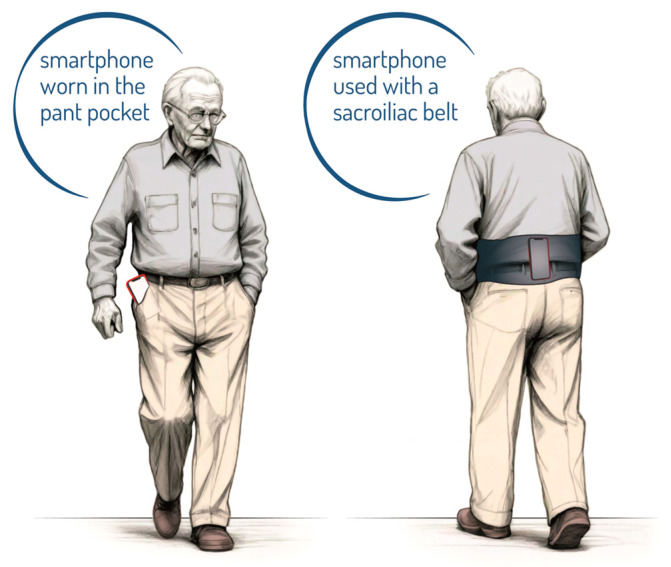
Smartphone placement: the corresponding position of the smartphone reported.

In this review, I did not find one particular smartphone set-up that was used by most studies, but a wide variety of combinations. The most used set-up was a smartphone attached to a sacroiliac belt, which was used in 8 of 11 studies screened [20,21,22,26,27,28,29,30]. The other position was in the pant pocket [14,23,25] (Figure 3).

Also, in terms of mobile operating systems, there was no homogeneity; 5 of the 11 studies screened used the Android operating system, and 6 of 11 used iOS. The G&B app for iOS represents the most used app, featured in 3 of 11 studies [21,26,28]. Most studies (6 of 11) reported the number of patients with a history of falls, with a maximum of 37 patients analyzed [20]; alternatively, other studies reported some motor/balance scores such as Berg Balance, the Kihon checklist score, the mini-balance evaluation system test, the functional gait assessment or the short physical performance battery. The heterogeneity of the scores used made it difficult to draw a conclusion and compare the functional ability of the sample recruited. Consensus still needs to be reached in this field; therefore, it is recommended that new studies report the history of falls in the analyzed sample.

### Data Analyses

Studies analyzed gait parameters collected with smartphones using descriptive statistics and ANOVA; analysis showed that age had a significant effect on smaller step frequency (*p* < 0.001) [20], worse step regularity and variability [29]. Gait cadence, which is also reported as step frequency, could be a useful indicator when estimating functional capacity, with a sensitivity of 0.81 (95% CI: 0.77, 0.85) and a specificity of 0.57 (95% CI: 0.55, 0.59) [27].

Other studies explored the test reliability and construct validity of mobile app data compared with clinical measurements (Time Up and Go Test). The sensitivity of app data demonstrated moderate-to-excellent reliability for walking looking straight ahead for gait symmetry (ICC = 0.65), walking speed (ICC = 0.93), step length (ICC = 0.94) and step time (ICC = 0.84) [21,30]. Giannouli et al. showed moderate-to-excellent test–retest reliability (ICCs between 0.584 and 0.920) and validity (ICCs between 0.468 and 0.950) of walking speed measurements of 50 m [25]. Olsen et al. recently confirmed moderate-to-excellent validity for mobile app measurements of step time (rp 0.97, 95% CI [0.96, 0.98]), walking speed (rp 0.83 [0.78, 0.87]) and step length (rp 0.74, [0.66, 0.80]) [28]. In contrast, not all spatiotemporal parameters had the same validity; in fact, Olsen et al. found that step length variability (r 0.29 [0.09, 0.47]), step length asymmetry (r 0.14 [0.06, 0.34]), step time variability (r 0.49 [0.31, 0.63]) and step time asymmetry (r 0.2 [0.01, 0.39]) had poor validity [26].

Three studies compared the smartphone app analysis with gold standard methods such as 3D kinematics, video assessment and the GAITRite^®^ system [14,26,28]. Lee P.-A. et al. showed a high correlation (r = 0.94), with minimal differences (mean = 0.07 m/s, ± 1.96 SD = 0.12) across a range of gait speeds and a high test–retest reliability (ICC values: 0.75 to 0.93) compared to video assessment analysis [14]. Results obtained by Olsen et al. showed excellent agreement between the mobile app and the GAITRite system for step time (rp 0.97, 95% CI [0.96, 0.98]), walking speed (rp 0.83, 95% CI [0.78, 0.87]) and step length (rp 0.74, 95% CI [0.66, 0.8]) [28].

## 4. Discussion

Biomechanical investigation allows the identification of abnormalities in gait that may also impact the quality of life and mobility of older adults [31]. One of the most important spatiotemporal parameters that seems to decline significantly in older patients is gait speed [21,22,32,33]. The emergence of technologies such as smartphones, mobile apps and artificial intelligence has provided promising avenues for gait analysis that had previously been performed in laboratories with a set of measurement systems such as stereophotogrammetry, EMG and force platforms [34]. Financial constraints and time expenditure have limited the use of these movement analysis laboratories in clinical practice; hence, the availability of cost-effective and reliable tools for gait analysis is paramount [35]. Gait analysis using smartphones and mobile apps is expected to play an increasingly important role in various clinical fields, where quantitative assessment of outcomes is crucial in achieving therapy goals [36,37,38]. Rehabilitation has significantly benefited from the development of smartphones and mobile apps [39]. In this context, quantifying the re-establishment of function is essential in the success of therapy, and this technology can be used to measure and monitor movements in order to support clinical decision-making [40,41]. Smartphones and mobile apps enable the objective and responsive assessment of physical function during functional tests, gait training or exercise programs, and the increasing use of smartphone technology in our daily lives and clinical settings will simplify patient assessment, therapy and follow-ups for health professionals [42]. Smartphones provide a cheap and accessible means of efficiently collecting large amounts of human gait data in an unconstrained environment compared to motion capture systems, electromyography or other systems that require costly equipment and trained engineers, which are only available in movement analysis research laboratories. To summarize, approaches to gait analysis in older individuals can vary regarding the type of mobile app, smartphone used, smartphone location and the type of spatiotemporal parameters assessed. The large number of patients analyzed in these studies (887 patients) suggests the feasibility of using a mobile app to quantify motor performance in older patients. This scoping review is the first to review articles concerning the application of smartphones to assessing spatiotemporal parameters of gait in older adults. Its novelty lies in providing current evidence regarding the effectiveness of gait assessment using mobile apps through smartphones in the aged population. The use of smartphones for gait analysis has been studied in patients with Parkinson’s disease, post-stroke and multiple sclerosis [43,44,45]. However, these patients have unique and variable gait patterns, so the results may not be applicable to the general elderly population. This study is not without limitations. One limitation of the present study concerns the inability to carry out a statistical analysis of results due to the heterogeneity of the control groups, mobile app used and type of gold standard chosen in order to investigate the validity of spatiotemporal gait parameters assessed with smartphones.

Consensus among the clinical research community regarding the smartphone’s location is yet to come. Looser pants with larger pockets may have led to more artifactual instrument movements and/or greater deviations from the participant’s center of mass, providing a poorer signal for data processing [46]. For this reason, the most reliable protocol to date is probably the one that involves positioning the smartphone using a sacroiliac belt. Despite the different mobile apps and smartphones used, all studies agreed on the reliability of these tools for measuring spatiotemporal parameters such as gait speed, the most investigated spatiotemporal parameter, in older adults, with excellent reliability [25,47,48,49].

## 5. Conclusions

This review provides strong evidence regarding the potential use of smartphone applications to assess gait impairments among older individuals. The results indicate that smartphone applications are tools with strong validity and reliability in monitoring gait dysfunctions, such as lower walking speeds and stride lengths, that are related to adverse outcomes, including disability and mortality. This study also highlights promising avenues for further research, emphasizing the importance of predictive modeling in addressing capability risks in the daily activities of the older population.

## Figures and Tables

**Table 1:** Characteristics of mobile apps, smartphones used and gait parameters: an overview of selected studies.

Reference	Experimental Group (N, Mean Age SD, Sex)	Patients with History of Falls	Mobile Phone App Used	Smartphone Used and Placement	Spatiotemporal Parameters Investigated	Main Findings
Zhong et al., 2020 [20]	148 participants (40 M/108 F) Age: 69.8 (7.0)	Yes. N = 37	Pocket Gait	Android smartphone (vivo Z1, Android operating system version 8.1, VIVO Technology Co, Dongguan, China) with a sacroiliac belt	Step symmetry, step frequency, RMS, step regularity and step variability	The Pocket Gait app is a health management tool that enables older adults to self-manage their gait quality and prevent adverse outcomes. The step frequency of participants in the age group 60 to 69 years was significantly higher than that of participants in the other age groups.
Shafi et al., 2023 [21]	83 participants(33 M/50 F) Age: 56.12 ± 6.06 years	Berg Balance Scale = 46 to 54, indicating a mild risk of falls	G&B	iOS. iPhone 7 used with a sacroiliac belt	Gait velocity, gait symmetry (periodicity index) (%), step time variability (%), average step length, average step time, step length variability (%), step length asymmetry (%) and step time asymmetry	The G&B app excels in specific areas, particularly in measuring walking speed, step length and step time, as emphasized by the alignment of these parameters with established clinical benchmarks and their moderate-to-excellent reliability. However, gaps remain, especially concerning the reliable assessment of steadiness, step length variability, step time variability, step length asymmetry and step time asymmetry.
Doshi et al., 2023 [22]	49 participants(7 M/42 F) Age: 75.6 (8.3)	Yes. N = 14	Lockhart Monitor	iOS. Apple smartphone used with a sacroiliac belt	Gait velocity	People with osteoporosis with a history of falls can be differentiated by using dynamic real-time measurements that can be easily captured using a smartphone app. Participants in the non-fall group walked faster (0.96 m/s) than those who had fallen (0.79 m/s).
Kawai et al., 2023 [23]	163 participants (104 M/59 F) Age: 72.1 (6.85)	n = 34 frailty group assessed using Kihon checklist score [24].	Authors customized a daily walking speed measurement app (Chami, InfoDeliver Co. Ltd., Tokyo, Japan)	Android smartphones worn in the pant pocket	Gait velocity, step length, cadence and number of steps	DWS measured using the smartphone application was associated with frailty. Step length was significantly smaller in the frailty group.
Giannouli et al., 2022 [25]	57 participants (27 M/30 F) Age: 75.3 (5.9)	Yes. N = 13	MOBITEC-GP	Android smartphones worn in the pant pocket	Gait velocity	The MOBITEC-GP app showed moderate-to-excellent test–retest reliability and validity of walking speed measurements.
Olsen et al., 2023 [26]	34 participants(14 M/20 F) Age: 42–94	No	G&B app	iOS. iPhone SE used with a sacroiliac belt	Gait velocity, periodicity index, mean step length, mean step time, step length variability, step time variability, step length asymmetry and step time asymmetry	The G&B app offers valid measurements of walking speed, step length and step time with a moderate-to-excellent reliability in older adults.
Rubin et al., 2022 [27]	37 participants(5 M/32 F) Age: 71 (69–74)	Yes. N = 8	Authors customized a Step Test application	iOS. iPhone 8 smartphone was placed in either the patient’s front pants pocket or attached to a waist belt	Cadence	The study demonstrates the feasibility of using gait cadence as a measure to estimate functional capacity.
Olsen et al., 2024 [28]	54 participants(20 M/30 F) Age: mean 61.6	No	G&B app	iOS. iPhone 7 or iPhone SE used with a sacroiliac belt	Gait velocity, mean step length, mean step time, mean left step length, mean right step length, mean left step time, mean right step time, step length variability, step time variability, step time, step length asymmetry and asymmetry	The G&B app has potential to provide valid measurements of step time, step length and walking speed in older adults.
Zhong et al., 2022 [29]	100 participants (56 M/44 F) Age: 73.0 (7.7)	Yes. N = 18	Pocket Gait	Android. Smartphone Huawei Honor v20 used with a sacroiliac belt	Step frequency (Hz), RMS acceleration (m/s^2^), step time variability, step regularity and step symmetry	The Pocket Gait app could be used to detect early signs of aging; older adults who walked less than 1 km had a lower quality gait compared with their counterparts who walked more than 1 km per day.
Lee et al., 2024 [14]	15 participants(15 F) Age: (77.67 ± 6.41)	No	Authors customized a smartphone application	Smartphones worn in the pant pocket	Gait speed	This mobile app has been shown to be valid and reliable for measuring gait speed in older adults and was highly correlated with video-based analysis.
Pedrero-Sánchez et al., 2023 [30]	65 participants (M/F: not reported) Age: 68.55 (7.18)	Yes. N = 25	FallSkip app	Android9. Smartphone Xiaomi Redmi 4 × Model MAG138 used with a sacroiliac belt	AP and ML displacement of the center of mass (CoM) during 30 s standing, vertical and ML excursion of the CoM while walking, average power of turning–sitting movements and standing up, range of AP jerk of CoM during turning–sitting movement and standing up, reaction time and total motion time	Fall risk can be reliably assessed using a simple, fast smartphone protocol that allows accurate fall risk classification among older people and can be a useful screening tool in clinical settings.

G&B app = Gait&Balance smartphone application; iOS = iPhone Operating System; DWS = daily walking speed; ML = medio–lateral; AP = anterior–posterior.

## Data Availability

Data supporting the reported results can be obtained by writing to lorenzo.brognara2@unibo.it.
